# Supplementary material for: Taste 2 Receptor Is Involved in Differentiation of 3T3-L1 Preadipocytes
Source: Int J Mol Sci. 2022 Jul 23;23(15):8120. doi: 10.3390/ijms23158120 (PMC9331636; doi:10.3390/ijms23158120)
Supplement: Supplementary file 1 [file ijms-23-08120-s001.zip › ijms-1833013-supplementary.pdf]

Supplementary Material

Taste 2 receptor is involved in differentiation of 3T3-L1 preadipocytes

Shunsuke Kimura [1,2], Ai Tsuruma [1] and Eisuke Kato [3,*]

[1] Graduate School of Agriculture, Hokkaido University, Kita-ku, Sapporo 060-8589, Hokkaido, Japan
[2] Japan Society for the Promotion of Science (JSPS), Chiyoda-ku, Tokyo 102-0083, Japan
[3] Division of Fundamental AgriScience and Research, Research Faculty of Agriculture, Hokkaido University, Kita-ku, Sapporo 060-8589, Hokkaido, Japan
* Correspondence: eikato@eis.hokudai.ac.jp

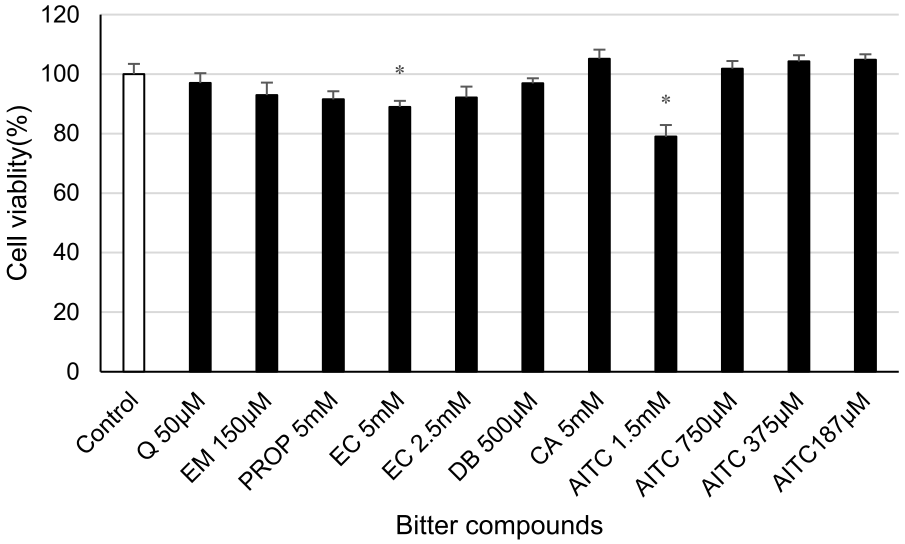

Figure S1. Cell viability after stimulation by bitter compounds. 3T3-L1 adipocytes are stimulated by bitter compound at the indicated concentration for 1 hr and cell viability was measured with Cell Counting Kit-8 (Dojindo Laboratories). Quinine (Q), emetine (EM), 6-propyl-2-thiouracil (PROP), epicatechin (EC), denatonium benzoate (DB), camphor (CA, 5 mM), allyl isothiocyanate (AITC).

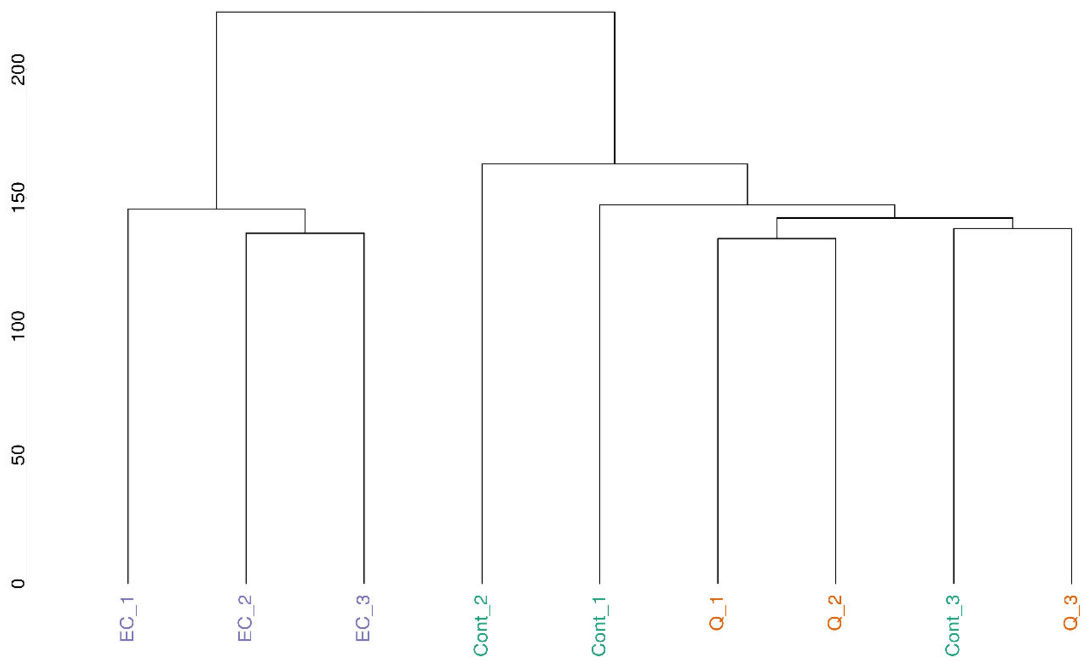

Figure S2. Clustering analysis of RNA-seq result. Cont_1-3: control cells. EC_1-3: epicatechin stimulated 3T3-L1 adipocytes. Q_1-3: quinine stimulated 3T3-L1 adipocytes.

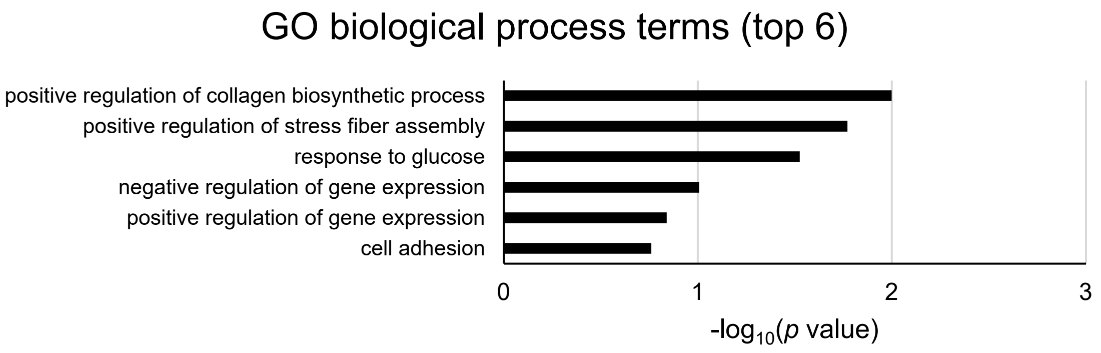

Figure S3. GO terms of downregulated genes of 3T3-L1 adipocytes stimulated by epicatechin.
